# Utility of patient information leaflet and perceived impact of its use on medication adherence

**DOI:** 10.1186/s12889-023-15346-y

**Published:** 2023-03-14

**Authors:** Majed Al Jeraisy, Heba Alshammari, Mashael Albassam, Kholoud Al Aamer, Mostafa A. Abolfotouh

**Affiliations:** 1 King Abdullah International Medical Research Center, King Saud Bin-Abdulaziz University for Health Sciences, Ministry of National Guard-Health Affairs, Riyadh, Saudi Arabia; 2 College of Pharmacy, King Saud Bin-Abdulaziz University for Health Sciences, Ministry of National Guard-Health Affairs, Riyadh, Saudi Arabia; 3 Pharmaceutical Care, Ministry of National Guard-Health Affairs, Riyadh, Saudi Arabia; 4 King Saud University Medical City, Riyadh, Saudi Arabia; 5 Saudi Food & Drug Authority, Riyadh, Saudi Arabia

**Keywords:** Perception, Attitude, Compliance, Package insert, Safe use of medicine, Drug labeling, Saudi

## Abstract

**Background:**

Although patients frequently use patient information leaflets (PILs) to obtain information about medicine, their confidence in using it may be diminished after reading it. This study aimed to assess the public perception of PIL's quality and the perceived impact of its use on medication adherence.

**Methods:**

A community-based cross-sectional study of 1,138 adult individuals in Saudi Arabia, April–May 2020, was conducted via Survey Monkey using an anonymous validated e-questionnaire. Data were collected on personal characteristics, PIL readership and preferences, perception towards PIL quality and impact of its use on taking medication, and reasons for not reading PIL. In addition, logistic regression analysis was performed to identify the significant predictors of reading PIL. Significance was considered at *p* < 0.05.

**Results:**

Nearly all participants (91.1%) reported reading PIL. The more read PIL's sections were directions of use (52.7%) and side effects (30.3%). Female gender (OR = 5.64, 95%CI: 3.53,9.02), age over 40 years (OR = 2.80, 95%CI: 1.69,4.64), and secondary education or more (OR = 1.74, 95%CI: 1.06,2.85) were the significant predictors of reading PIL. The majority of PIL readers reported their preference for verbal information (65.8%), hard copy presentation (77%), adding graphics (71.1%), and concise content of PIL (68.8%). In addition, most participants reported PIL always/usually adds to their knowledge of medicines (70.6%) and said that PIL reading positively impacted their medication adherence (64.9%). For only 8.8%, PIL reading negatively impacted their adherence, primarily because of reading information on medicine's side effects and complications (74.4%). More than one-half of participants perceived the PIL quality as good/excellent in terms of; font size (51.3%), language comprehensiveness (64.9%), paper quality (68.0%), and general appearance (64.9%). Getting sufficient information from doctors and pharmacists was the main reason for not reading the PIL (59.2%). Most participants (92.5%) agreed on standardizing how information is displayed in the PIL among all PILs of all companies.

**Conclusion:**

PIL is read by nearly all the study sample, especially females, older, and educated subjects. It was perceived as beneficial in upgrading medication adherence. Effective designing of PILs should focus on patients' literacy level and age. Standardization of the PIL structure in all pharmaceutical companies is recommended.

**Supplementary Information:**

The online version contains supplementary material available at 10.1186/s12889-023-15346-y.

## Background

Several medications, especially over-the-counter (OTC) medications might look safe from the patients’ point of view. Misusing these drugs, such as increasing the dosage or taking inappropriate medicines, may have significant risks and numerous complications like drug intoxication, drug interactions, allergy, and teratogenicity [1]. A cross-sectional study of 390 respondents conducted in a Saudi Arabian city found that using non-prescribed medications was practiced by more than three-quarters of the study sample [2]. The community has become more aware of the risks of medicines, the ease of access to the internet, and the presence of smartphone applications made it easy to reach information at any time. Since some websites and applications provide inaccurate or false information about medications, it became more important to have a complete and relevant reference attached to these medications to ensure patient safety and drug efficacy [3, 4].

### Systems of medication information provision

There are two international systems to provide medication information. First, the Package Insert (PI) follows the United States Food and Drug Administration (USFDA) guidelines and provides information for patients and healthcare professionals. In January 2006, the USFDA issued final regulations regarding (PI) referred to as the "Physician Labeling Rule" (PLR). It aims to promote the safety and effectiveness of f prescription drug product use by providing precise and concise PI that is easier to access, read, and use to health care providers [5, 6]. Second, the patient information leaflet (PIL), released by the European Medicines Agency (EMA) and is focused on patient information and is intended mainly for patient use, and the Summary of Product Characteristics (SPC), which is intended for health care professionals. Both systems have online access to drug information [7–9].

### PIL as a source of medication information in the Arab world

PIL would be an appropriate reference that includes all the information needed by the patient. It is a summary of drug information written by drug manufacturing companies. It is a crucial source of information, and it is considered a reliable and visible reference for the medications of the patients and health care providers to answer their medication-related queries [10, 11]. In Saudi Arabia, irresponsible self-medication was expected, and physicians and pharmacists were the most common source of information (80.2%), followed by PIL (58%) [12]. Medication use and side effects were the most common information that participants were interested in [12]. Other Studies in the United Arab Emirates (UAE) showed that PILs were a significant source of drug information for healthcare professionals and an essential pharmaceutical company marketing tool [13].

### Utility of PIL in Saudi Arabia

The utility of PIL, in terms of reading and understanding, represents one of the causes of medication errors and poor adherence [14, 15]. A study of the effect of PIL on patient behavior in Israel showed that the rate of reading the PIL was about 50% and that reading PIL aroused anxiety and decreased adherence in some patients [16]. Sixty PILs were evaluated in a study conducted in Saudi Arabia. It was reported that the current PIL style needs to be improved to avoid medication errors and ensure the safe and appropriate use of medications [10]. Despite efforts to improve the readability and comprehensibility of drug regulatory authorities and manufacturers, package inserts still needs to be criticized [17–20]. This is mainly due to the extensive volume of incomprehensible text and the small font size used, which people find distressing [20, 21].

### PIL in the Arab world in comparison with Western countries

The Saudi Food and Drug Authority (SFDA) adopted the EMA for the drug information pamphlet since it has two categories of medication information, one for healthcare providers (SPC) and the other one for patients' consumers (PIL). PIL provides information regarding medication indication, serious and possible side effects, and instructions for usage and storage. Each PIL should be reviewed every three years or when necessary [22]. A survey of a Saudi-based cross-sectional study about Public attitude toward technical drug packages [done by over 2000 community pharmacy customers found that 88% of respondents claimed they read the PIL or asked somebody to read it for them [23]. A study done by Bawazir et al. [24], to compare the Saudi-marketed products and the US drug labeling reported that the PIL of Saudi-marketed drugs contained limited and incomplete information compared with their counterparts sold in the USA. Another study to assess the PIL for generic medicines manufactured in the Middle East and Saudi Arabia showed that information in the PIL of the marketed drug was different from generic package inserts of the British National Formulary [25].

### PIL readership rates

Readership rates in some countries have been reported to range from 40 to 97% [16, 26–33], and comprehension of the leaflet’s content was variable [30, 34–37]. However, no recent Saudi studies provide such statistics. A previous study to examine the quality of PIL in Saudi Arabia [10] showed that information related to the safety and appropriate use of drugs was not stated in the PILs. Our study aimed to assess the utility of PIL by medication consumers in a Saudi population through the following: (1) Assessment of the readership rate of PIL by the Saudi public, (2) Assessment of the perceived quality of PIL and perceived impact of its use on medication adherence, and (3) Determination of the reasons for not reading the PIL.

## Methods

### Study design

A community-based cross-sectional survey of the Saudi public on the utility of PIL was conducted.

### Study population

Our study population included a convenient sample of the public in different cities of Saudi Arabia. The sample respondents included Saudi citizens of both gender, who can read PIL in the Arabic or English language, and aged ≥ 18 years at the survey date. People who were not included in our study were health care providers (pharmacists, Physicians, Nurses), people who lacked internet access, or people who were not able to complete an online survey.

### Data collection

For the physical distancing strategy and to minimize face-to-face interaction, we developed an online questionnaire [38, 39] via Survey Monkey (https://www.surveymonkey.com) that limits 1-time participation per unique internet protocol (IP) address. This questionnaire was designed to be sent to a convenient sample of the public in different cities of Saudi Arabia from April–May 2020 via groups on Facebook, Twitter, Instagram, etc. Inclusion criteria were; Saudi citizens of both gender who can read PIL in the Arabic or English language and aged ≥ 18 years at the survey date. Exclusion criteria were; Health care providers (pharmacists, Physicians, Nurses), lack of access to the internet, or inability to complete an online survey.

A previously validated questionnaire was adopted and modified [38, 39]. It consisted of 21 questions regarding the following topics [The complete questionnaire is added in Supplementary material]:

- Personal characteristics such as; patient's sex, age, level of education, chronic illness, and sources of medication (government hospital, private hospital, community pharmacies),
- PIL readership: Ever reading the PIL(s), frequency and time of reading, reasons for reading, and the part of the PIL that is frequently read,
- Preferences of PIL characteristics: Information type, presentation, adding graphics, and quality of content,
- Perception towards PIL and its impact: Influence of PIL reading on the way of taking the medication, and reasons for not reading the PIL(s).
- Perceived quality of PIL, in terms of; clarity of font size, language comprehensiveness, paper quality, and general appearance, using a scale of 1–4, 1 = poor, 4 = excellent, and
- Reasons for not reading the PIL: Participants were asked to choose one or more from the following reasons; side effects and complications, interactions with other medications or food, the remedy is inappropriate for their condition, and allergy from the drug or one of the excipients.

The reliability of the Arabic version of the questionnaire was assessed by distributing it to 70 participants to check for clarity of questions and identify any misunderstandings. Data from this pilot study was not included in the final analysis. In addition, we have assessed the questionnaire in terms of internal consistency. Cronbach's alpha was computed, and a coefficient alpha of 0.84 was considered adequate. Test–retest reliability was also assessed using Cronbach's alpha and Pearson's correlation coefficient (r). Construct validity of the checklist was assessed using expert opinion, and the final version was approved after making the necessary modifications.

#### Sampling technique

Based on the assumption of a PIL readership rate of 40% [25], and with a margin of error of 0.03 and 95% confidence limits, the sample size of 1024 was estimated. To compensate for an average of 50% non-response to the e-questionnaire and incomplete data collection, a sample size of ~ 2,000 was adopted. Those who responded with completed questionnaires were 1,138 respondents.

#### Data analysis

Data entry and statistical analysis were performed with the Statistical Package for Social Science (SPSS) software program for Windows (version 25.0). Descriptive statistics, such as frequencies, means, and standard deviations, were calculated. A chi-square test was applied for categorical data, and unpaired two-sample t-test and ANOVA were applied for continuous data. Univariate analyses were performed to determine the association between PIL readership and the following independent variables; age (≤ 40 years versus > 40 years), gender, education (secondary or less versus more than secondary), comorbidities (yes versus no), and source of medication (government hospital, private hospital, community pharmacies). Logistic regression analysis was performed to determine the significant predictors of PIL readership. Crude and adjusted odds ratios and corresponding 95% confidence intervals were calculated. Statistical significance was considered at *p* < 0.05 for all analyses.

#### Outcomes measures

(1) Rate of ever PIL readership, (2) Perceived quality of PIL, (3) Level of the perceived impact of PIL use on medication adherence, (3) Preferences of PIL characteristics, and (4) Reasons for not reading the PIL(s).

## Results

### Sample characteristics (Table 1)

**Table 1:** Demographic characteristics of the study sample

Variables	No	%
*Gender*		
Male	412	36.2
Female	726	63.8
*Age (in years)*		
18–25	221	19.3
26–40	470	41.1
41–60	414	36.2
> 60	39	3.4
*Education*		
High school	176	15.5
Diploma	133	11.7
Bachelor Degree	680	59.8
High Degree	133	11.7
Other	15	1.3
*Comorbidities*		
Yes	477	58.3
No	667	41.7
*Source of Medication*		
Government hospital	393	34.4
Private hospital	317	27.8
Community pharmacy	432	37.8

Figures in the table are for only available data

A total of 1,138 subjects responded with completed questionnaires to the survey. The majority (63.8%) were females, ages 26 to 40 years (41.1%), had a bachelor's degree (59.8%), and had one or more chronic comorbidities (58.3%). The source of getting medicines was distributed equally among community pharmacies (37.8%), governmental (34.4%), and private hospitals (27.8%), Table 1.

### Patients' use of PIL (Table 2)

**Table 2:** Patients’ use of PIL and preferences to improve its readership

	**No**	**%**
*Have you ever read PIL?*		
Yes	1041	91.0
No	103	9.0
*How often do you read PIL?*		
Always/usually	585	56.4
Sometimes	378	36.4
Rarely/never	75	7.2
*When?*	No	%
With every new prescription	628	60.4
In all medication	135	13.0
If side effects	86	8.3
If I forgot	191	18.4
*Why?*		
To find specific information on side effects	595	57.4
To get other information about the drug	441	42.6
*Which section?*		
Ingredient	11	1.1
Dosage	53	5.1
Side effect	315	30.3
Uses	548	52.7
Contraindication	98	9.4
Storage	15	1.4
*How do you prefer to get information?*		
Verbally from Dr./Pharm	749	65.8
Written	390	34.2
How do you prefer reading the PIL		
Hard copy	799	77.0
Website	63	6.1
Via smart device	176	17.0
*How do you prefer the depth of information in PIL?*		
Concise	716	68.8
Detailed	324	31.2
*Do you prefer adding graphics?*		
Yes	739	71.1
No	301	28.9
*Do you agree on standardizing how information is displayed in the PIL?*		
Yes	960	92.5
No	78	7.5

Figures in the table are for only available data

Of all surveyed subjects, 91% reported reading PIL, and nearly one-half said they always read PIL, with every new prescription, to either find a specific information on side effects or to get medication information. The more read PIL sections were directions of use (52.7%) and side effects (30.3%). The majority of PIL readers prefer verbal information from physicians or pharmacists (65.8%), reading PIL as a hard copy (77.0%), concise PIL (68.8%), and added graphics (71.1%). The majority agreed on standardizing how information is displayed in the PIL among all PILs of all companies (92.5%), Table 2.

### Predictors of reading PIL among the participants (Table 3)

**Table 3:** Predictors of PIL readership among the participants

	**no(%)**	**cOR (95% CI)**	***p*** **-value**	**aOR (95% CI)**	***p*** **-value**
*Gender*					
Male	340 (82.5)	1^a^		1^a^	
female	695 (95.7)	1.16(1.12–1.22)	< 0.001*	5.64 (3.53–9.02)	< 0.001*
*Age*					
≤ 40 years	613 (88.7)	1^a^		1^a^	
> 40 years	428 (94.5)	1.07 (1.03–1.10)	0.001*	2.80 (1.69–4.64)	< 0.001*
*Education*					
Secondary or less	162 (84.8)	1^a^		1^a^	
More than secondary	875 (92.5)	2.02 (1.35–3.03)	0.001*	1.74 (1.06–2.85)	0.0027*
*Comorbidities*					
Yes	432 (90.6)	1^a^		1^a^	
No	609 (91.3)	1.01 (0.97–1.05)	0.67	0.8 (0.5–1.2)	0.224
*Source of medicine*					
Governmental hospital	353 (89.8)	1^a^		1^a^	
Private hospital	295 (93.1)	0.97 (0.92–1.01)	0.13	1.26 (0.71–2.23)	0.43
Community pharmacies	391 (90.5)	0.99 (0.95–1.04)	0.11	1.14 (0.69–1.89)	0.60

Figures in the table are for only available data

^a^Reference category

*Statistical significance, *cOR* crude odds ratio, *aOR* adjusted odds ratio, *CI* confidence interval

In bivariate analyses, PIL readership was significantly associated with female gender (OR = 1.16, 95%CI:1.12–1.22), older age of > 40 years (OR = 1.07, 95%CI: 1.03–1.10), and secondary or more education (OR = 1.09, 95% CI: 1.02–1.16). Even after adjusting for possible confounders in a logistic regression analysis, female gender (OR = 5.64, 95%CI: 3.53–9.02), age of over 40 years (OR = 2.80, 95%CI: 1.69–4.64), and secondary or more education (OR = 1.74, 95% CI:1.06–2.85) were the significant predictors of PIL, Table 3.

### Perception towards the quality of PIL and impact of its use (Table 4)

**Table 4:** Perception of the participants towards the quality of PIL and its impact on knowledge on medication and medication adherence

Responses	No	%
*How frequent does PIL add to your knowledge?*		
Always	395	38.0
Usually	339	32.6
Sometimes	253	24.3
Rarely	28	2.7
Never	25	2.4
*Have reading PIL impacted your way of taking medication?*		
No impact	269	26.3
Increase adherence	664	64.9
Decrease adherence (why?)	90	8.8
• Side effects/ complications	67	74.4
• Interactive with other drugs	19	21.1
• Inappropriate for my condition	27	30.0
• Allergy from drug	19	21.1
Perceived quality of PIL	Poor/fair No.(%)	Good/excellent No. (%)
• Font size	131 (48.7)	138 (51.3)
• Language comprehensiveness	93 (35.1)	171 (64.9)
• Paper quality	83 (32.0)	177 (68.0)
• General appearance	93 (35.1)	166 (64.9)

Figures in the table are for only available data

Most of PIL readers reported that PIL reading always/usually adds to their knowledge of medicines (70.6%). Meanwhile, more than two-thirds said that PIL reading positively impacted their medication adherence. For only 8.8%, PIL reading impacted their adherence negatively, primarily because of reading information on side effects or medicine complications (74.4%). The majority of participants reported good/excellent quality of PIL in terms of; font size, language, paper quality, and general appearance, Table 4.

### Reasons for not reading PIL (Fig. 1)

**Fig. 1:**
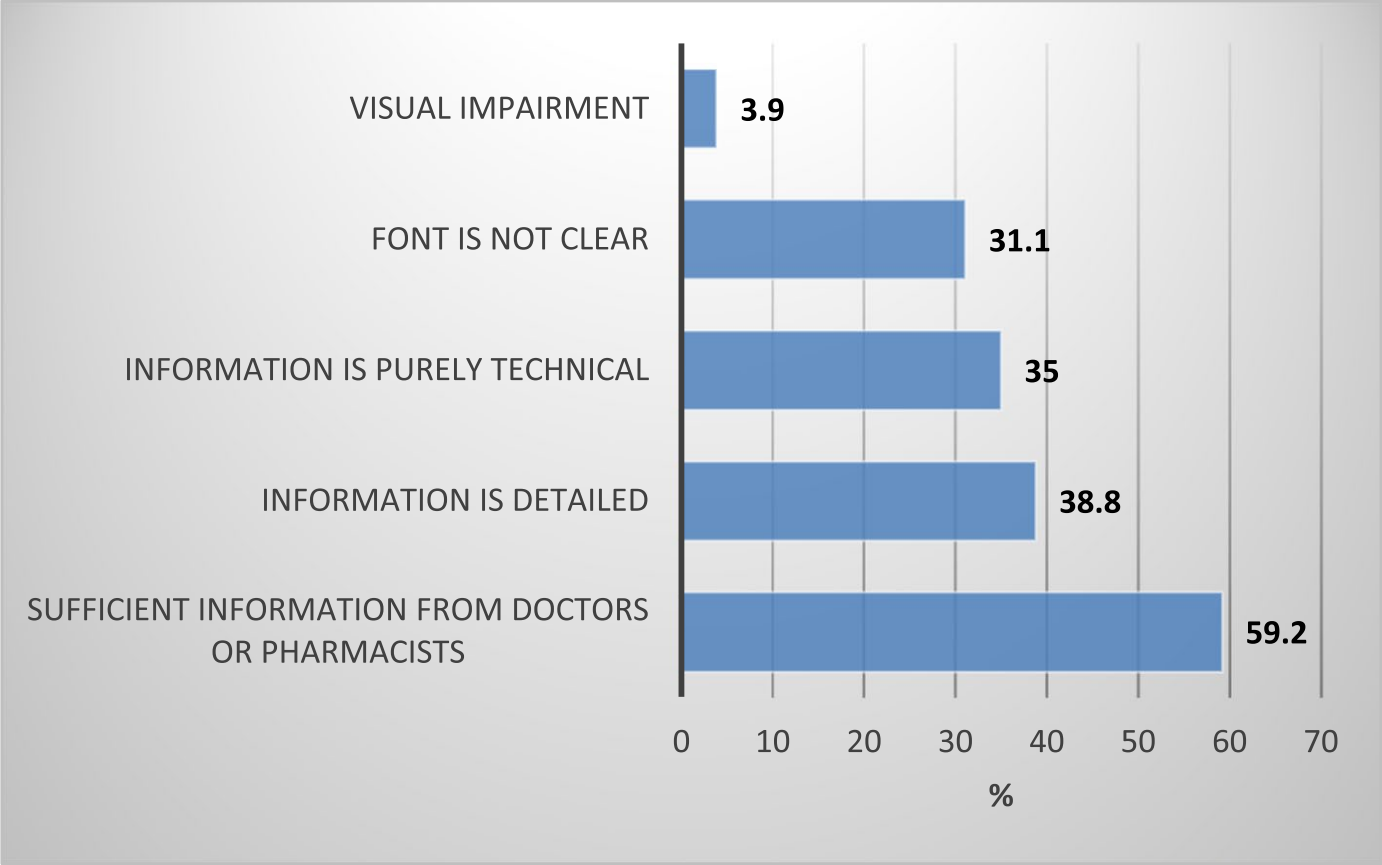
Reasons for not reading the medication information leaflets Note: Categories are not mutually exclusive

Figure 1 shows that more than one-half (59.2%) of participants reported that sufficient information they got from doctors and pharmacists was the reason for not reading the PIL. Other reasons were because they see PIL information as more detailed (38.8%), more technical (35.0%), or because the font needs to be more clear.

## Discussion

This study aimed to assess the public perception of PIL's quality and the impact of its use on medication adherence. Nearly all participants reported reading the PIL; the more read PIL's sections were directions of use and side effects. Female gender, age over 40 years, and secondary education or more were the significant predictors of reading the PIL. Most of PIL readers reported a preference for verbal medical information, hard copy presentations with graphics, and the concise content of PIL. The information written on the PILs was readable and understandable by literate subjects.

### Readership rate

The utility of the leaflet depends first on the extent to which it is read and second on the degree to which patients understand it. PIL readership rate varies among other countries such as Saudi Arabia (88%) [23], Iran (84%) [40], Turkey (78.2%) [32], Palestine (74.3%) [11], and Israel (51.5%) [16]. In our study, nearly all subjects claimed they read the PIL. However, when they were asked to report how often they read it, only one-half of all PIL readers reported reading it always, one-half reported reading it with every new prescription, one-half reported reading it to either find a piece of specific information on side effects or to get more medication information. In Palestine, 45 percent of consumers reported they always read the PIL, and 29.3% said they read the PIL most of the time [11]. The more read PIL sections were directions of use (read by one-half) and side effects (read by one-third). These results justify the finding of previous studies that patients who read the PIL are more likely to follow the medication instructions [41].

Information given verbally may need to be misunderstood or remembered. That is why a paper label is given [42]. In our study, most PIL readers prefer verbal information from physicians or pharmacists rather than reading it from PIL. However, the preference for PIL as concise, hard copy with added figures (pictures) ranked first by the most PIL readers. It had been reported that patients of a lower socioeconomic status requested more verbal information on their treatment [43]. A study comparing the effect of oral and written information on patients with mild mental retardation found that the written statement was less helpful and even confusing to some patients [36]. In a previous study in Pakistan, nearly one-half of the surveyed patients were found to be keen on reading inserts in their mother language [44]. In a Palestinian survey [11], the preferred language for the PILs was Arabic for most consumers. At the same time, it was English for most healthcare professionals, and most customers and health professionals reported the need to improve of information in the PILs. In our study, nearly all participants agreed to standardize how information is displayed in the PIL among all PILs of all companies. These findings show that the style and design of PILs must be tailored to the targeted population.

### Predictors of readership

Published reports about the association between patients' demographic characteristics and reading a PIL are controversial. Females tended to read the PIL more than males in some studies [11, 45, 46]. This was in agreement with the findings of our study. Many studies revealed that females are less likely to take risks than males [47, 48]. However, other published studies did not report any significant association between gender and the likelihood of reading the PIL [16, 26, 40, 49]. Regarding age, some studies have not shown any association with reading a PIL [11, 16, 26, 40]. It has been reported that the elderly tend not to use modern drug information sources and rely primarily on interpersonal contact with healthcare personnel [46]. Another previous study reported a negative association between age and reading PIL [50]. However, our study showed that reading PIL was significantly more among those over 40 years. Paying particular attention to the elderly by healthcare workers is recommended to ensure medication use safety.

In the present study, the chronicity of comorbid conditions was not associated with the reading of PIL. This finding was in agreement with the results of previous studies [11, 16, 40]. However, In Israel [16], patients tended to read the PIL for chronic medications more than those for acute therapy. This may be due to the perceived risk of long-term treatment and the need for more information on the drugs prescribed and their possible adverse effects. Many studies have reported education as a significant predictor of PIL readership [16, 40, 51, 52]. In the US, the literacy level suggested to design the PIL was in the 6th–8th reading levels [53]. We believe that in Saudi Arabia, we need to standardize to help with more effective PILs, given the Saudi population’s low level of health literacy as reported in previous studies [54, 55], where the majority of Saudi individuals had inadequate health literacy associated with poor knowledge of health information. If the Saudi FDA mandates drug companies to comply with a standardized format for PIL, these companies have to abide by this policy.

### Perception of PIL impact

The physician or the pharmacist talks about the usefulness of the PIs can create a positive feeling encouraging the patient to read the PIL [51]. However, in a previous study, only 8% of patients were encouraged to read the PIL by their healthcare workers [52]. In our study, most participants reported PIL reading always/usually adds to their knowledge of medicines. This finding was in agreement with the results of other studies [11, 26, 27, 40]. In our study, one-fourth of PIL readers reported no change in their behavior in taking medications. In contrast, the majority (two-thirds) reported that PIL reading had positively affected their adherence to medicine, and less than 10% reported a negative impact. This finding was the opposite of the results reported in a previous study in the US [26], where three-quarters of participants indicated that they did not change, and only one-quarter said that they did. This discrepancy might be attributed to the difference in perception of PIL quality. In our study, nearly one-half of the participants perceived the PIL quality as good and excellent in font size, language, paper quality, and general appearance. In contrast, the other half perceived it as poor and fair. This finding was discordant with a previous USA study, where most patients reported that leaflets were valuable and easy to understand [26]. However, a direct comparison between that study and ours is difficult because of the different study designs and assessment tools used.

Confidence in using the medicine by the patients may be diminished after reading the PIL. Experience of fear of taking the medication after reading the side effects in PIL has been reported [27, 40, 45, 56, 57], leading to non-adherence in the form of discontinuing the medication prematurely or changing the dose of the medicine without the physician's approval [58, 56]. Fortunately, in our study, only 8.8% of PIL readers reported that PIL reading impacted their adherence negatively, primarily because of reading information on side effects and medicine complications. This finding was in agreement with a previous study in Israel, where treatment adherence was decreased by 9.7% [16]. However, Gibbs et al. [31] found no increase in adverse effects of drugs when information was provided. Seeking information about the side effects of the medication was reported as the main reason for reading the PIL [26, 27, 40, 58, 45, 59]. In our study, direction for use was the most common section read by patients, followed by the side effects section. This finding agreed with a previous study in Saudi Arabia [23], where the general public emphasized the importance of indications and adverse drug reactions mentioned on PILs. In a prior study in Iran [40], about one-fourth of patients stated that they would store the PILs as a drug information source. In our study, 8% reported reading PIL if side effects occur. However, medication error and poor patient compliance may be the consequences of problems in reading and understanding PIL by the patients [60].

### Reasons for not reading the PIL

The three most commonly reported reasons were that; sufficient information was provided by the physician and the pharmacist, the leaflet needed to be shorter and more detailed, and the font needed to be clearer. These findings were in agreement with the results of a previous USA study [26]. The first most commonly reported reason could justify the conclusions of an overall positive perception and attitude toward community pharmacists and high satisfaction with pharmacists' commitment and communication skills in a recent survey in Saudi Arabia [61], contrary to the findings of western countries, where pharmacists did not adequately counsel a substantial number of patients, and many aspects of drug therapy were not sufficiently discussed [61–64]. The second most commonly reported reason for not always reading the PIL was that it needed to be shorter, more detailed, and technical. This finding emphasizes the importance of producing concise PIL. Lack of clarity in the PIL font was the third reason for not reading the PIL. In the present study, in agreement with the findings of a previous study, about one-fourth of respondents faced problems reading and understanding PIL [44]. These findings reflect the necessity for more improvements in the style and design of PIL and improvement in the contents.

### Strengths and limitations

This study may provide significant results that help introduce information on the Saudi public perception of PIL's quality and the impact of its use on medication adherence to the literature. In addition, it may act as a pilot study to other ones from similar countries. The study's strength lies in its large sample size, recruited during a crucial period—the early stage of the COVID-19 outbreaks in Saudi Arabia when pharmacies remained open to serve customers through governmental assigned online delivery applications and systems. Yet, people might rely on reading the PIL to take the medications rather than consulting physicians who were not accessible all the time during the Covid-19 curfew. Moreover, participants in this study were representative of all the regions of Saudi Arabia.

However, this study has some limitations: The survey was conducted using social media (WhatsApp, Twitter, and Facebook), and we cannot predict the response rate. Moreover, the study excluded people who lacked access to the internet, which could result in selection bias. Furthermore, the study may be subjected to recall bias, as the data were self-reported. In addition, the cause-and-effect relationship is not guaranteed due to the cross-sectional design. Thus, it is difficult to determine whether the exposure (age, sex, education level, etc.) or outcome (PIL readership) came first. Another limitation was that other parameters affecting the likelihood of reading the PIs were not measured in this study, such as health literacy, occupational status, patient coping style, and health status. Further studies could assess this point. However, these results may give baseline data about our country's situation, which meets this study's aims.

## Conclusion

Generally speaking, our study showed that although the leaflet may be an essential source of information, many patients use it only partially. This highlights the importance of adequate medication counseling in ensuring the proper use of medications. In addition, PIL needs to be improved in terms of readability and understanding, considering the patient demographics, especially education level and age. The results of this research would help policymakers and health authorities improve the format and availability of PILs. Saudi FDA and all related authorities should take action, in collaboration with pharmaceutical companies, to update the guidelines for PILs’ quality and clarity, using the guidelines of western countries as a reference. Pharmacists should ensure that the leaflet is personally handed to the patient rather than placed in or attached to the medication. They should also review the PIL with patients and advise them to use it as a reference. Future studies are necessary to investigate the association between the quality of PIL and the utility of its use.

## Supplementary Information

**Additional file 1.**

## Data Availability

Most of the data supporting our findings is contained within the manuscript, and all others, excluding identifying/confidential patient data, will be shared upon request, by contacting the corresponding author [Mostafa Abolfotouh mabolfotouh@gmail.com].

## Abbreviations

PILs: Patient information leaflets
OTC: Over the counter
PI: Package Insert
USFDA: United States Food and Drug Administration
PLR: Physician Labeling Rule"
EMA: European Medicines Agency
SPC: Summary of Product Characteristics
SFDA: Saudi Food and Drug Authority
IRB: Institutional Review Board
MNGHA: Ministry of National Guard-Health Affairs
UAE: United Arab Emirates
