# Supplementary material for: Utility of patient information leaflet and perceived impact of its use on medication adherence
Source: BMC Public Health. 2023 Mar 14;23:488. doi: 10.1186/s12889-023-15346-y (PMC10012310; doi:10.1186/s12889-023-15346-y)
Supplement: Supplementary file 1 — Additional file 1. [file 12889_2023_15346_MOESM1_ESM.docx]

**QUESTIONNAIRE**

**Utility of Patient Information Leaflet and Perceived Impact of its Use on Medication Adherence**

This survey is part of a research that aims to study the perception of Saudi community toward patient information leaflet.

The study population is Saudi society members (18 years old and above). The data will be collected through this self-administered survey. It will take about 5 minutes to complete. Be assured that all answers will be kept confidential.

*Thank you for agreeing to take part in our survey. You can fill the survey only one time

*The study was approved by the IRB at King Abdullah International Medical Research Center (KAIMRC) under protocol # RC20/098/R

| Consent answer | Do you agree to participate in this questionnaire? | - Yes, I Agree - No, I do not agree |
| --- | --- | --- |
| Question 1 | Do you work in medical field? | - Yes - No |
| Question 2 | Where do you get your medication? | - Governmental hospital. - Privet hospital. - Community pharmacies |
| Question 3 | Do you suffer from any medical condition of the following? (you can choose more than one answer) | - Diabetes - Hypertension - Heart disease - Psychological and mental disorders - Renal/hepatic disorders - Autoimmune disorders - Oncology disorders - Respiratory disorders - I do not suffer from any disease - Other |
| Question 4 | Which way you prefer to get information about your medications? | - Verbally from the doctor/pharmacist - Written in a paper |
| Question 5 | Have you ever read the Patient Information Leaflet (PIL)? | - Yes - No |
| Question 6 | Why have not you read the PIL? (you can choose more than one answer) | - The font not clear - I get sufficient information from doctor or pharmacist - Visual impairment - The written information purely scientific for specialists - The written information detailed in unsuitable manner - Other |
| Question 7 | How often do you read the PIL? | - Always - Usually - Sometimes - Rarely - Never |
| Question 8 | When do you read the PIL? | - With every new prescribed medication - In all medication you take - If you notice any side effect - If you forgot an information about the medication |
| Question 9 | Why do you read the PIL? | - To find out a specific information like side effects. - To get more information about the medication |
| Question 10 | Which part of the PIL you are keen to read? | - Ingredients/excipients - Dosage - Side effects - Uses of the medication - Contraindications - Storage conditions |
| Question 11 | Is reading the PIL adds to your knowledge an information you are looking for? | - Always - Usually - Sometimes - Rarely - Never |
| Question 12 | How do you prefer the depth of the information in the PIL? | - Brief - Detailed |
| Question 13 | How do you prefer reading the PIL? | - Hard copy - Website - Application of smart devices |
| Question 14 | Have reading the PIL influenced your way of taking the medication? | - Yes - No |
| Question 15 | What was the impact? | - It increased my compliance to take medicine properly - It increased my fears and decrease the adherence to take the medication |
| Question 16 | If reading the PIL has led to decrease your compliance or prevented you from taking the medication, choose one of the following reasons (you can choose more than one answer) | - Side effects and complications - Interactions with other medications or food - The medication is inappropriate for your condition - Allergy from the drug or one of the excipients |
| Question 17 | The following section evaluates PIL in terms of clarity and appearance. (using a scale of 1-4, 1= poor, 4 = excellent) |  |
| 17-1 | Font size | - 1 - 2 - 3 - 4 |
| 17-2 | Level of language comprehensiveness | - 1 - 2 - 3 - 4 |
| 17-3 | Quality of paper/printing | - 1 - 2 - 3 - 4 |
| 17-4 | General appearance | - 1 - 2 - 3 - 4 |
| Question 18 | Do you prefer adding graphic illustrations to PIL? | - Yes - No |
| Question 19 | Do you agree on standardized how information are displayed in the PIL among all PILs of all companies? | - Yes - No |
| Question 20 | Which authority is responsible for the monitoring and approval of PILs? | - Ministry of Health - Saudi Food and Drug Authority - Pharmaceutical companies - I do not know |
| Question 21 | *Demographics*  Your Age | - 18-25 years old - 26-40 years old - 41-60 years old - Older than 60 years |
| Question 22 | Your Gender | - Male - Female |
| Question 23 | Your current Province | - Riyadh - Mecca - Medinah - Alqassim - Eastern region - Assir - Tabuk - Hail - Northern Borders Region - Jizan - Najran - Albaha - Aljouf |
| Question 24 | Your Qualifications | - High school - Diploma - Bachelor’s degree - Higher degree - Other |
| Question 25 | University or professional major |  |
| Question 26 | Suggestions: Write your suggestions for improving the contents of the PIL |  |
